# Supplementary material for: Identification of photoperiod-induced specific miRNAs in the adrenal glands of Sunite sheep (Ovis aries)
Source: Front Vet Sci. 2022 Jul 22;9:888207. doi: 10.3389/fvets.2022.888207 (PMC9354845; doi:10.3389/fvets.2022.888207)
Supplement: Supplementary file 1 [file Data_Sheet_1.ZIP › Supplementary file/miRDeep2 file.zip!/miRDeep2 file/Know_Structure/LP42A2/oar-mir-99a.pdf]

miRBase precursor : oar-mir-99a
Total read count : 536905
oar-miR-99a read count : 536043
remaining reads : 862

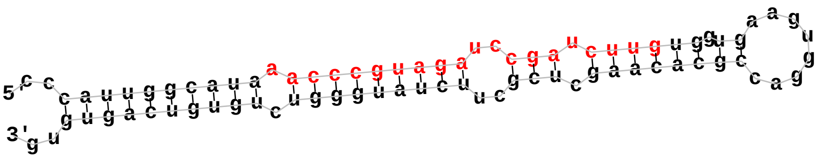

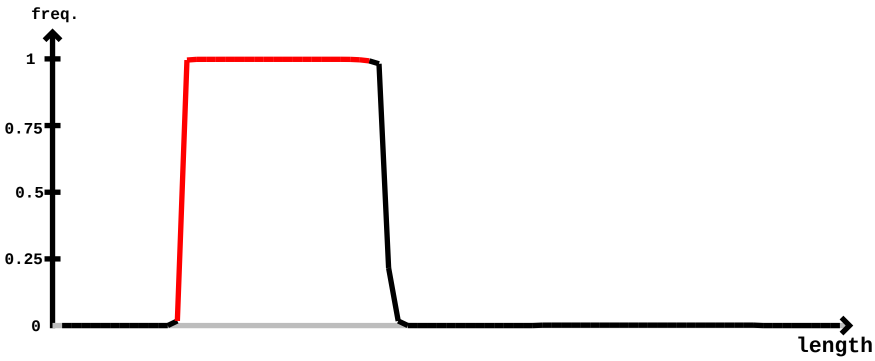


oar-miR-99a

```
5'- cccauuggcauaaacccguagauccgaucuuguggugaaguggaccgcacaagcucgcuucuaugggucugugucagugug   -3'    exp
    ..((((((((((.(((((((((..(((.((((((.((........)))))))).)))..))))))))).))))))))))..   reads  mm  sample
    ..........uaaacccguagauccgaucuu..................................................   2      0   seq
    ..........uaaacccguagauccgaucuugu................................................   2      0   seq
    ...........aaacccguagauccgauc....................................................   2      0   seq
    ...........aaacccguagauccgaucu...................................................   17     0   seq
    ...........aaacccguagauccgaucuu..................................................   35     0   seq
    ...........aaacccguagauccgaucuug.................................................   76     0   seq
    ...........aaacccguagauccgaAcuugu................................................   2      1   seq
    ...........aaacccguagauccgaucuugu................................................   9007   0   seq
    ...........aaacccguagauccgaAcuugug...............................................   1      1   seq
    ...........aaacccguagauccgaucuugug...............................................   209    0   seq
    ...........aaacccguagauccgaucuugugg..............................................   14     0   seq
    ............aacccguagauccgaAc....................................................   2      1   seq
    ............aacccguagauccgauc....................................................   139    0   seq
    ............aacccguagauccgaAcu...................................................   107    1   seq
    ............aacccguagauccgaucu...................................................   754    0   seq
    ............aacccguagauccgaAcuu..................................................   158    1   seq
    ............aacccguagauccgaucuu..................................................   1995   0   seq
    ............aacccguagauccgaucuug.................................................   4791   0   seq
    ............aacccguagauccgaAcuug.................................................   915    1   seq
    ............aacccguagauccgaAcuugu................................................   21101  1   seq
    ............aacccguagauccgaucuugu................................................   380172 0   seq
    ............aacccguagauccgaucuugug...............................................   77268  0   seq
    ............aacccguagauccgaAcuugug...............................................   28553  1   seq
    ............aacccguagauccgaucuugugg..............................................   8836   0   seq
    ............aacccguagauccgaAcuugugg..............................................   382    1   seq
    ............aacccguagauccgaAcuuguggu.............................................   6      1   seq
    ............aacccguagauccgaucuuguggu.............................................   21     0   seq
    .............acccguagauccgaAcuu..................................................   2      1   seq
    .............acccguagauccgaucuu..................................................   4      0   seq
    .............acccguagauccgaAcuug.................................................   12     1   seq
    .............acccguagauccgaucuug.................................................   6      0   seq
    .............acccguagauccgaucuugu................................................   436    0   seq
    .............acccguagauccgaAcuugu................................................   221    1   seq
    .............acccguagauccgaucuugug...............................................   108    0   seq
    .............acccguagauccgaAcuugug...............................................   544    1   seq
```

oar-miR-99a

```
cccauuggcauaaacccguagauccgaucuuguggugaaguggaccgcacaagcucgcuucuaugggucugugucagugug
.............acccguagauccgaucuugugg..............................................    11    0    seq
.............acccguagauccgaAcuugugg..............................................    41    1    seq
.............acccguagauccgaAcuuguggu.............................................    1     1    seq
..............cccguagauccgaucuugu................................................    14    0    seq
..............cccguagauccgaucuuguggu.............................................    1     0    seq
...............ccguagauccgaucuugu................................................    31    0    seq
...............ccguagauccgaAcuugu................................................    3     1    seq
...............ccguagauccgaucuugug...............................................    4     0    seq
...............ccguagauccgaAcuugug...............................................    4     1    seq
................cguagauccgaucuugu................................................    24    0    seq
................cguagauccgaucuugug...............................................    8     0    seq
................cguagauccgaucuugugg..............................................    1     0    seq
................................................acaagcucgcuucuaugggucugu.........    1     0    seq
.................................................caagcucgcuucuauggg..............    1     0    seq
.................................................caagcucgcuucuauggguc............    1     0    seq
.................................................caagcucgcuucuaugggucu...........    5     0    seq
.................................................caagcucgcuucuaugggucug..........    20    0    seq
.................................................caagcucgcuucuaugggucugu.........    782   0    seq
.................................................caagcucgcuucuaugggucugug........    3     0    seq
.................................................caagcucgcuucuaugggucugugu.......    2     0    seq
..................................................aagcucgcuucuaugggucug..........    1     0    seq
..................................................aagcucgcuucuaugggucugu.........    43    0    seq
..................................................aagcucgcuucuaugggucugug........    1     0    seq
...................................................agcucgcuucuaugggucug..........    1     0    seq
...................................................agcucgcuucuaugggucugu.........    1     0    seq
```
